# Optimised feature selection-driven convolutional neural network using gray level co-occurrence matrix for detection of cervical cancer

**DOI:** 10.1515/biol-2022-0770

**Published:** 2023-11-30

**Authors:** K. Sudhakar, D. Saravanan, G. Hariharan, M. S. Sanaj, Santosh Kumar, Maznu Shaik, Jose Luis Arias Gonzales, Khursheed Aurangzeb

**Affiliations:** Department of Computer Science & Engineering, Madanapalle Institute of Technology & Science, Madanapalle, Andhra Pradesh, India; Department of Computer Science and Engineering, Sathyabama Institute of Science and Technology, Chennai, Tamil Nadu, India; Department of Artificial Intelligence and Machine Learning, Malla Reddy University, Hyderabad, India; Department of Computer Science and Engineering, Adi Shankara Institute of Engineering and Technology, Kalady, Ernakulam, Kerala, India; Department of Computer Science, ERA University, Lucknow, Uttar Pradesh, India; Department of ECE, Vidya Jyothi institute of Technology, Aziznagar, Hyderabad, India; Universidad Tecnologica de los Andes, Abancay, Peru; Department of Computer Engineering, College of Computer and Information Sciences, King Saud University, P. O. Box 51178, Riyadh 11543, Saudi Arabia

**Keywords:** cervical cancer detection, convolutional neural network, accuracy, gray level co-occurrence matrix, feature selection, pep test images

## Abstract

Cervical cancer is one of the most dangerous and widespread illnesses afflicting women throughout the globe, particularly in East Africa and South Asia. In industrialised nations, the incidence of cervical cancer has consistently decreased over the past few decades. However, in developing countries, the reduction in incidence has been considerably slower, and in some instances, the incidence has increased. Implementing routine screenings for cervical cancer is something that has to be done to protect the health of women. Cervical cancer is famously difficult to diagnose and cure due to the slow rate at which it spreads and develops into more advanced stages of the disease. Screening for cervical cancer using a Pap smear, more often referred to as a Pap test, has the potential to detect the illness in its earlier stages. For the purpose of selecting features for this article, a gray level co-occurrence matrix (GLCM) technique was used. Following this step, classification is performed with methods such as convolutional neural network (CNN), support vector machine, and auto encoder. According to the findings of this experiment, the GLCM-CNN classifier proved to be the one with the highest degree of precision.

## 1 Introduction

Cervical cancer is one of the most dangerous and widespread illnesses afflicting women throughout the globe, particularly in East Africa and South Asia. It is also one of the most common cancers in women. In 2008, around 530,000 new cases of obstructive cervical cancer were detected in women all over the world. As a direct result of these diagnoses, 275,000 women lost their lives to the disease. There is a significant disparity in the distribution of the disease burden across different nations due to the fact that developing countries are responsible for 86% of all cervical cancer diagnoses and 88% of all cervical cancer deaths worldwide [1]. There is a higher than average prevalence of cervix cancer in India, which accounts for one-quarter of the global total prevalence rate. This disease is present in more than 80% of the world’s countries. In recent years, the proportion of women in industrialised nations who have been given a diagnosis of cervical cancer has significantly decreased. This decline may be traced back to the direct outcome of vigorous public education programmes [2]. In industrialised nations, the incidence of cervical cancer has consistently decreased over the past few decades. However, in developing countries, the reduction in incidence has been considerably slower, and in some instances, the incidence has actually increased.

An imbalance in the rate of cell proliferation in the cervix is the primary contributor to the development of cervical cancer. Cervical cancer is an extremely rare kind of cancer. Cancer that develops in the cervix has the risk of becoming deadly. The lowest section of the uterus, often known as the belly, is referred to as the cervix in medical terminology. This structure is also known as the cervix of the uterus from time to time. It is located at the end of the uterus. The uterus, sometimes referred to as the “womb,” is the organ that is responsible for the development of an embryo into a newborn (upper half). During pregnancy, the cervix serves as a structural link between the vaginal area and the uterus. This connection is essential for the development of the baby. This connection must exist in order for the baby to be delivered successfully (birth trench). The term “endo-cervix” refers to the portion of the cervix that is located in close proximity to the inside of the uterus and is located within the cervix. There are two types of cells that may be seen on the surface of the cervix: epithelial cells and squamous cells. On the exocervix, squamous cells account for around 50% of the entire mass of the cervix. In the human body, there is an area known as the change zone that is located at the point where two separate populations of cells meet and interact with one another. The transition zone is the part of the cervix where the great majority of instances of cervical cancer originate. Malignancies that originate in the cervical area often begin in the cervix’s epithelium as the first stage of development. They do not mysteriously transform into cancerous cells all of a sudden for no apparent cause. The formation of cancer is the end consequence of precancerous modifications that take place in the normal cells of the cervix [3,4].

**Figure 1:**
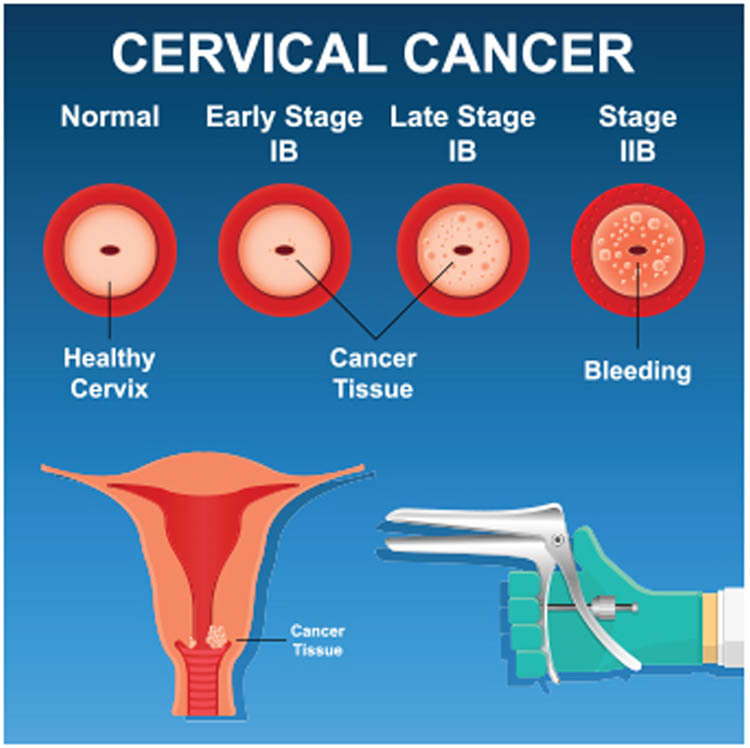
Cervical cancer stages.

It is an example of a risk factor that could have a role in determining the possibility of one being afflicted with a disease such as cancer. Cancer comes in many distinct forms, and each of those forms has its own unique set of risk factors. Spending a lot of time in the sun may increase the likelihood that you may acquire skin cancer at some point in your life. People who smoke have an increased risk of developing a number of different forms of cancer. Even if a person has just one risk factor, this does not always suggest that they will get cancer as a direct result of that factor alone. When many risk factors are present, there is an increased likelihood of developing cervical cancer. When none of these risk factors are present, a woman has a very low chance of developing cervical cancer. Even while possessing these risk factors significantly increases a woman’s probability of developing cervical cancer, there are still a significant number of women who do not end up having the disease. If a woman is diagnosed with cervical cancer or pre-cancerous abnormalities in her cervix, it is possible that they will not be connected to a single risk factor with a certainty of 100%. Everyone who can quit the habit of smoking or prevent themselves from being infected with the human papillomavirus (HPV) is considered a member of this group (HPV). In spite of this, it is essential to take into consideration the risk factors that cannot be altered. This is for the simple reason that it is much more vital for women who have these risk factors to have frequent Pap tests to detect cervical cancer at an earlier stage. The stages are shown in Figure 1.

According to the World Health Organization (WHO), death rates and incidence rates for cervical cancer vary from country to country [5,6]. In 2008, cervical cancer was responsible for the deaths of 275,000 women all over the globe. Eighty-eight percent of the deaths took place in poor nations, with Asia being responsible for 159,800 of those deaths. When discussed in the context of medicine, cervical cancer is sometimes described as an infection of the singularity. According to the findings of the recent study, the created world has a greater rate of cervical cancer, which also has a higher mortality rate. According to the Crisis Card, the greatest rate of death in the world may be found in Africa. The number of women in Australia who have died due to cervical cancer is at an all-time low. One reason for this is the widespread use of HPV antibodies, treatment, and public expectations.

In the United Kingdom, cervical cancer accounts for around 2% of all newly diagnosed instances of cancer in females, placing it as the tenth most prevalent illness in females. In 2010, 2,851 women in the United Kingdom were diagnosed with cervical cancer. According to the yearly incidence rate, there are around nine newly diagnosed cases of cervical cancer for every one hundred thousand females in the United Kingdom. In this computation, unprocessed incidence rates are utilised. The International Agency for Cancer Research estimates that yearly 275,000 women lose their lives as a result of cervical cancer. In addition, it is the most common cause of mortality from cancer in women all over the globe (266,000 deaths in 2012) [7,8].

To safeguard the health of women, it is imperative that tests for cervical cancer become standard practice and become part of regular healthcare. A big additional barrier to the physical and mental well-being of modern women is the manner in which they live their life, the food that they consume, and the way that they behave in the current society. Due to the sluggish pace at which it spreads and develops into more severe stages of the illness, cervical cancer is notoriously difficult to identify and treat until it has progressed to an advanced stage. The screening for cervical cancer known as a Pap smear, which is more often referred to as a Pap test, offers the ability to identify the condition in its earliest stages. There is a multi-step process that must be completed before one may detect anything and then decide what that detection means. It is imperative that you get the advice of an expert before deciding on a certain plan of action. Doing so will ensure that you make the best decision possible. Experience is much more significant than intellectual aptitude when it comes to determining the best course of action to take in a given situation. To be able to determine if a slide of pap smear results includes positive or negative cells, one has to possess the specialised knowledge of microscopy as well as the capacity to put that information into practice. Due to the fact that there are seven different kinds of cells, the task at hand is far more difficult than it would be otherwise if there were just two different kinds of cells.

Image processing algorithms have come a long way in recent years, which has been of considerable help to modern machine vision systems. On the other hand, machine learning has made possible a whole new set of approaches to the resolution of problems. In circumstances in which the application of human thought is unable to solve a problem, a learning algorithm may be used to train a computer on how to discover a solution on its own to solve the issue. This is the objective that is sought for by the machine vision field. It is possible that the rule-based systems that were in existence in the past, which were formed via the use of hand-crafted tools and processes and were based on characteristics that were observed, were able to sort through obstacles and arrive at conclusions. On the other hand, the program does not cope with potentially dangerous situations since it is unable to react to them or discover a solution to them. Because of this, adaptive learning would not be possible until computers are able to learn from their own mistakes. Until then, it would not be possible. On the other hand, the term “machine learning” refers to the process by which a computer imitates human intelligence by learning about a problem and various aspects of the problem’s underlying knowledge to use that information to solve issues in real time and arrive at a final solution to the problem. In other words, a computer mimics human intelligence by learning about a problem and various aspects of the problem’s underlying knowledge. The term for this kind of activity is “machine learning.”

Gray level co-occurrence matrix (GLCM) is used to extract features. GLCM shows space and grey size distribution in an area. This example uses only four of many texture options. ECH – energy, contrast, correlation, and homogeneity – is their acronym. The photo shows these features. When analysing stochastic textures, the GLCM is typically utilised. This version improves the image and adds explanation. A photo can be analysed using the GLCM metric with various pixel brightness values. For instance, the co-occurrence matrix shows that two pixels might appear in several spatial orientations based on their distance and angular spatial relationships. Image texture, colour, contrast, and shape are obtained using the GLCM. Texture analysis is very important in machine learning and human visual perception. It emphasizes key diagnostic system components to demonstrate its reliability. GLCM texturing is vital for image processing and used frequently.

Deep learning is reliant on the representation of data acquired from the real world so that it can automate the learning process. This dependency is for the aim of making deep learning more efficient. It is more challenging to design specialised software when a picture comprises additional items in addition to the one that has to be found. This is because there are more things for the program to take into consideration. To find anything inside an image, one has to have a broad notion of where that object is positioned in relation to the rest of the picture. This is necessary to find anything. There is a possibility that the deep learning technique, which is one that makes use of neural networks (NNs), will be used here. The concept of “deep learning” has been receiving a significant amount of attention in the mainstream media as of late.

For the purpose of selecting features for this article, a GLCM technique was used. It is possible to obtain high-level information about a picture by employing the GLCM, such as the image’s texture, colour, contrast, and shape. In addition, both human visual perception and machine learning make extensive use of texture analysis as an important component in their respective processes. It is a useful instrument for demonstrating the reliability of the diagnostic system by directing attention to the various essential parts of the system. The textural feature that goes by the name GLCM is one that is vital for image analysis and is utilised quite frequently. Following this step, classification is performed with methods such as convolutional neural network (CNN), support vector machine (SVM), and auto encoder. The results of this experiment show that GLCM-CNN had the highest accuracy of all the classifiers that were examined.

## 2 Literature survey

Alyafeai and Ghouti [9] discovered and differentiated malignant spots in cervical pictures by making use of a classification strategy that relied on deep learning. Through the use of this approach, cancerous regions were identified. Deep learning is the term that describes this kind of instruction. CNNs were used by researchers to recognise photographs of the cervical region to make a cancer diagnosis. Morphological processes were used so that the cancerous parts of cervical pictures could be identified and segmented. In this particular instance, cancerous cells taken from the cervix were utilised. The authors’ ground truth images were accurate 97.2% of the time, 98.3% of the time, and 98.5% of the time, according to the Guanacase Dataset 2005.

CNNs are a type of artificial intelligence that can be used to classify cervical cell segmentations. Allehaibi [10] presented the Mask R-CNN, which is a visual geometry group-like network with a more condensed training set (VGG-like Net). The network that makes up the Mask R-neural CNN is constructed on top of ResNet10, which gives it the ability to make use of both spatial input and information that it has previously learned. The data from the Herlev Pap Smear are utilised to validate our methodology. The preceding segmentation technique is surpassed by Mask R-CNN in terms of performance metrics such as accuracy (0.910.05), recall (0.910.05), and ZSI (0.91).

According to Gupta et al. [11], for instance, data mining has the potential to be used to assist in the prevention of cervical cancer. Researchers were able to narrow down the potential risk factors for the development of cancer in patients by using the Boruta analysis. All of the demographic, historical, and medical aspects of this study are taken into account in this research. The “Hospital Universitario de Caracas” in Caracas did not make the “missing values” in this dataset of 858 patients available to the general public because the hospital wanted to protect the patients’ right to privacy. The construct of an evaluation of the same thing may be constructed using the criteria that were supplied, provided that they are relevant.

Treatment of the virus that causes the sickness was shown to be an effective technique for preventing the development of cervical cancer in patients, according to Deswal et al. research [12]. Because there are many different ways in which cervical cancer may spread, there are also many different strategies to diagnose and treat it at an early stage.

Karthiga Jaya and Senthil Kumar [13] identified and isolated regions of photos harbouring cervical cancer by using the artificial neural network fuzzy inference system (ANFIS) classification approach. Researchers were able to classify cervical photos by making use of an image registration strategy and an ANFIS classification method. Both of these techniques matched non-correlated photographs and made it possible for them to be categorised for cancer diagnosis. Therefore, it is feasible to detect and separate the cervical malignant patches using the morphological methodologies that are utilised to categorise images of cancer’s effects. When the authors’ methodology was compared against images taken on the ground, the Guanacase Dataset 2005 was used, which resulted in the discovery of an accuracy rate of 99.12%. The authors’ methodology had an average sensitivity, specificity, and accuracy rate of 97.42%.

Anousouya Devi et al. [14] looked at photographs of the cervical region by doing calculations based on the structure of the nervous system. The developers of the plan connected their computation to the district development division plan so that the results would be more accurate when applied to their proposed cancer division.

It was proposed by Kumar and Srivastava [15] that a method may be established to determine the reason why the cervixes of certain women are covered with unusual growths. The cervical regions of the patients were scanned via computer-aided detection, which stands for computer-assisted detection. There is potential for improvements in the accuracy of cervical cancer diagnosis to be made via the use of soft computing and computer-aided methods. When compared to the photographs that served as the ground truth, they found that their method had a sensitivity of 86% and a tumour segmentation accuracy of 91%.

Pap smear cell screening was shown to be the most effective method for detecting cervical cancer in female patients in a study that was conducted by Chen et al. [16]. On the image of the cervical region, an SVM classifier was utilised to extract information on the picture’s surface characteristics and lifelikeness. The authors had a Pap smear cell classification percentage of 96.12% on average across all of their samples. As a direct consequence of this, the identification of the great majority of cells was successful. You are going to want to use this procedure with high-quality photographs to get the most out of it.

The categorisation of cervical pictures was accomplished by the use of feature extraction, which was carried out by Mouelhi et al. [17]. The authors were successful in obtaining information on the texture and gradient of both healthy and diseased populations. These attributes were trained and classified with the help of subfold classifiers in preparation for their usage in machine learning applications.

When trying to detect whether a patient had cervical cancer, Bergmeir et al. [18] utilised a classification system that was based on feature extraction. To train the texture and energy characteristics that were obtained from the cervical picture, they used a technique that is known as quasi supervised learning. In the dataset that was utilised to evaluate the effectiveness of the proposed method, cervical photographs that had previously been seen as well as those that had not been seen before were included. Based on the 132 cervical photos, there were only 19% that were considered to be false positives, which resulted in an 88.0% true positive rate.

Classifiers based on SVM were created by Huang and Lai [19] for the early diagnosis of cervical cancer. In the beginning, the researchers began by using image analysis tools to extract textural characteristics from histopathology photos. These attributes were used in the training and classification processes of the SVM classifier. Researchers had a success rate of 92.8% when it came to accurately classifying instances of cervical cancer.

The innovative method that Chen et al. have devised [20] makes it feasible to perform cell segmentation in the cervical regions of patients. In the light of the radiologist’s previously segmented nucleus areas, the characteristics of the nucleus that had been segmented were analysed. By adhering to the procedures that the authors advocated, the authors were able to achieve an overall accuracy in categorisation that was 93% on average.

Researchers looked at numerous different classification approaches, such as the one developed by Rama Praba and Ranganathan [21], to determine how to detect and classify cervical pictures. Researchers enhanced cervical imaging and classified the acquired textural information using SVM and NNs. Within the scope of this investigation, extracted characteristics and NN classification were combined to accomplish the goal of achieving the highest possible classification accuracy of 89%.

## 3 Methodology

**Figure 2:**
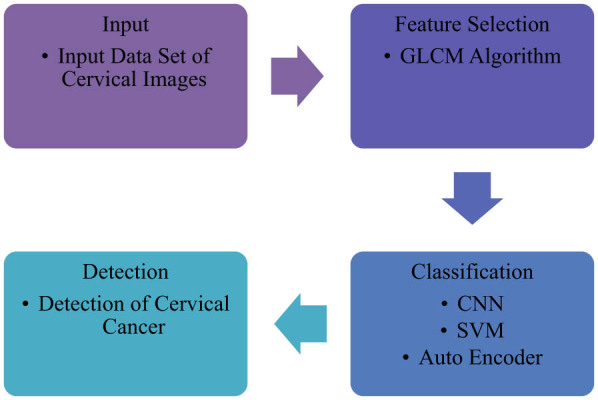
Optimised feature selection-driven CNN using gray level co-occurrence matrix for detection of cervical cancer.

This section presents optimised feature selection-driven CNN using GLCM for the detection of cervical cancer. It is shown in Figure 2. The GLCM approach for feature selection is included in this methodology section. After that, classification is carried out using techniques such as CNN, SVM, and auto encoder.

To extract features, GLCM [22] is used. The GLCM is a representation of the space and grey size distribution in a certain location. Only four of the many different texture possibilities are used in this particular illustration. In summary, they are referred to as ECH, which stands for energy, contrast, correlation, and homogeneity. These traits may be gleaned from the photo. The GLCM is one of the strategies that is used rather often when stochastic textures are being analysed (GLCM). In this version, the image has been improved, and the explanation has been included. When analysing a photo using the GLCM metric, there are several distinct permutations of the pixel brightness values that may be used. According to the calculation of the co-occurrence matrix, for instance, two pixels may simultaneously exist in a variety of spatial orientations with regard to the distance and angular spatial interactions between the two pixels. The GLCM is used to get high-level information about an image, such as its texture, colour, contrast, and shape. In addition, both human visual perception and machine learning make significant use of texture analysis as a key component. It is an effective tool for showing the soundness of the diagnostic system by focusing attention on important components. The texture characteristic known as GLCM is an essential one for image analysis and is used rather often.

Auto-encoders are a kind of unsupervised NN that attempts to copy input to output. Auto encoders are used in datasets to cut down on the total number of dimensions included in the data. AEs are constructed by using NNs’ encoder and decoder layers. The encoder layer is the one that is responsible for taking in the image data points. The encoder function will use this input to produce a hidden representation space denoted by the letter z. Encoding and decoding a Gaussian distribution allows for the calculation of the distribution’s mean as well as its variance. Because of this, a decoder takes the latent representation into consideration as an input and then generates distribution parameters for the data points that are chosen from the input [23,24].

It is possible to use SVM to classify both linear and nonlinear data. It does this by first using a nonlinear mapping to elevate the original training data up a dimensionality inside this new dimension. After doing this, it searches for the linear optimal separation hyper plane and decision boundary that divides tuples of one class from those of another class. An example of a constructive learning algorithm is the SVM, which is based on statistical learning theory and the Vapnik Chervonenkis dimensions. This strategy, which is founded on the principle of structural risk reduction, places its major emphasis not on the square of the mean square error over the whole dataset but rather on the generalisation error. Because of this mistake, the likelihood of these mistakes having a significant impact on the situation is significantly increased. If the SVM is able to generalise well to data that were not included in the training set, then it will have a good performance when it is applied to data that were not included in the training set. A clear and succinct explanation for the learned model was found as a direct consequence of employing the SVM. Even if the data used for training SVMs are biased, the models’ flexibility and resilience will continue to improve. Elements in the training set that are located close to or inside the decision limits of the classifier are referred to as support vectors. The classifier is able to form its decision lines thanks to the use of these support vectors in the decision-making process. The classifier is able to differentiate between normal and abnormal by making use of examples that are on the verge of falling into either category. A hyperplane may be used to construct SVMs by partitioning many data sets in the appropriate manner. Because hyper planes are created, the SVM is able to recognise the boundaries between input class categories. The establishment of these boundaries is the responsibility of the individual bits of input data that are together referred to as support vectors.

It is a common practise for computer vision technologies to make use of CNN, which is a kind of architecture for a deep NN. A CNN is composed of many layers, the most important of which are the convolutional, activation, pooling, and connected convolutional layers. Each convolutional layer of a deep CNN is linked to the subsequent layers in the network and so on. The filters in the convolutional layer are the most important component of that layer. A very small number of pixels from the input picture, maybe only three by three in size, are subjected to the filter that the convolutional layer employs [25].

The pixel values are processed by the filter using a dot operation, and the weights for each dot are supplied individually. To obtain a single output that is representative of all of the pixels that were entered into the filter, the sum of all of the weights is added together and utilised. As a direct result of this, the picture that is produced by the convolutional layer has a lot smaller matrix of data points than it had when it was first created. After the activation layer, which offers nonlinearity, has been provided to the activation layer, backpropagation is used to train the network. Backpropagation is used to train the network.

Sample production is cut down due to the layer-down sample pooling technique, which also results in the matrix of the filter being of a smaller size. It is referred to as the “max layer” when the pooling layer chooses the most essential feature from each group and then picks that characteristic. Calculating the label probabilities of an image may be accomplished by utilising the output of a linked layer that is connected to the maximum layers of the picture. The classification is done based on the response that is most likely to occur.

## 4 Result analysis and discussion

The Guanacaste collection was used to obtain the cervical photographs that were analysed for this inquiry [26]. This dataset has been annotated by experienced radiologists with images that depict the actual findings of each cervical scan. These photographs represent the ground truth. The investigation makes use of a total of 250 photographs. A model is initially taught with 175 unique photos, and then it is evaluated with 75 unique photographs. This component of the methodology includes a way for selecting features called the GLCM. By using this method, you can choose which characteristics to use. A GLCM [22] is utilised to extract features from a given dataset. A depiction of the space and grey size distribution in a particular area can be found in the GLCM. Following this step, classification is performed with methods such as CNN, SVM, and auto encoder. Encoder and decoder layers, which are found in NNs, are utilised in the construction of AEs. A CNN, often known as a CNN, is made up of multiple layers, the convolutional, activation, pooling, and connected convolutional layers being the most crucial ones. Every single convolutional layer that makes up a deep CNN is connected to the layers that come after it in the network and so on. The filters that make up the convolutional layer are the most vital part of that layer.

Four parameters such as accuracy, sensitivity, specificity, and precision are used in this study to compare the performance of different algorithms.

- Accuracy
  The proportion of all predictions that are predicted correctly (accurately) is referred to as accuracy.
\[{\mathrm{Accuracy}}\left=({\mathrm{TP}}\left+{\mathrm{TN}})\left/({\mathrm{TP}}+{\mathrm{TN}}+{\mathrm{FP}}\left+{\mathrm{FN}}).]\]
- Sensitivity
  The proportion of positive cases projected as positive is known as sensitivity. It is also known as “Recall.”
\[{\mathrm{Sensitivity}}\left=\left({\mathrm{TP}})\left/({\mathrm{TP}}\left+{\mathrm{FN}}).]\]
- Specificity
  The proportion of actual negative cases projected as negative is known as sensitivity.
\[{\mathrm{Specificity}}\left=\left({\mathrm{TN}})\left/({\mathrm{TN}}\left+{\mathrm{FP}}).]\]
- Precision

\[{\mathrm{Precision}}={\mathrm{TP}}\left/({\mathrm{TP}}\left+{\mathrm{FP}}),]\]

where TP is true positive, TN is true negative, FP is false positive, and FN is false negative.

**Figure 3:**
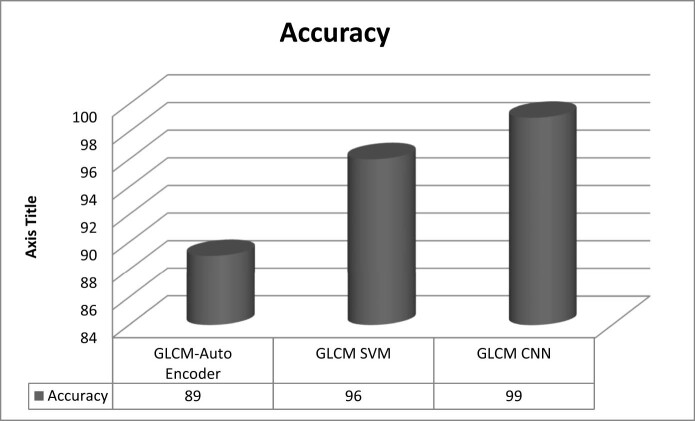
Accuracy of classifiers for cervical cancer detection.

**Figure 4:**
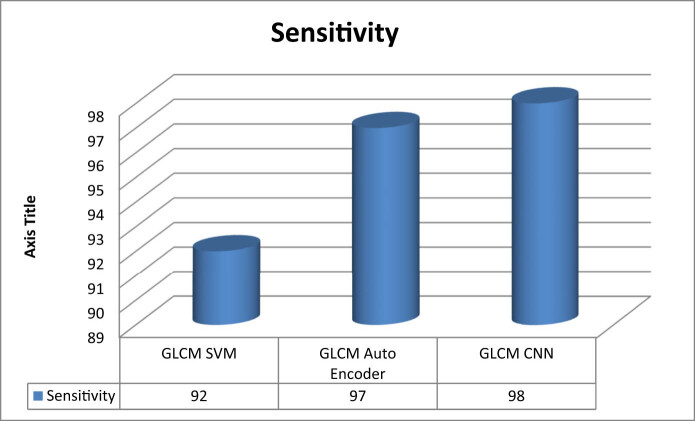
Sensitivity of classifiers for cervical cancer detection.

**Figure 5:**
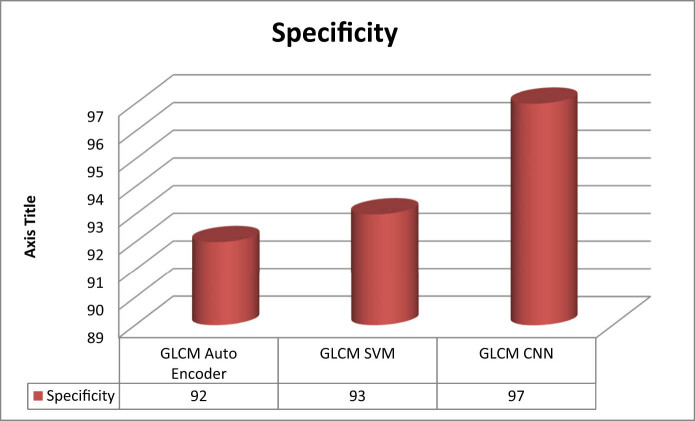
Specificity of classifiers for cervical cancer detection.

**Figure 6:**
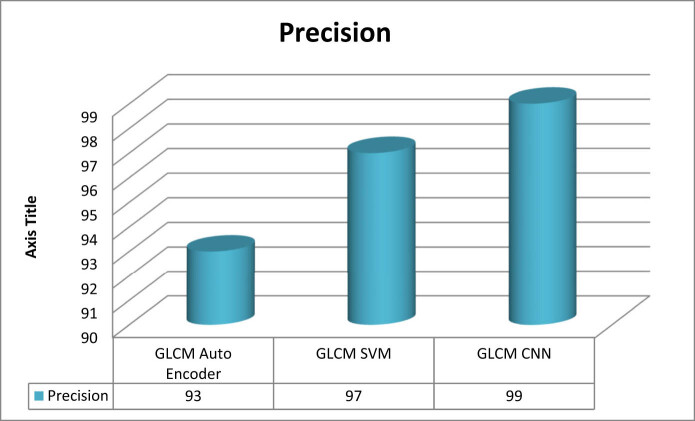
Precision of classifiers for cervical cancer detection.

Figures 3–6 illustrate the accuracy, sensitivity, specificity, and precision of the GLCM-SVM, GLCM-auto encoder, and GLCM-CNN in detecting cervical cancer. The GLCM-CNN classifier had the highest accuracy of all the classifiers that were utilised in this experimental investigation. The accuracy of the GLCM-CNN has been measured at 99%; this is 3% better than the accuracy of the GLCM-SVM and 10% better than the accuracy of the GLCM-auto encoder. The sensitivity of the GLCM-CNN is also 98%, which is a larger percentage than the sensitivity of the GLCM-auto encoder and the GLCM-encoder combined. The specificity of the GLCM-CNN is 97%, which is higher than the specificity of both the GLCM-auto encoder and the GLCM-CNN. The GLCM-CNN, the GLCM-auto encoder, and the GLCM-SVM all have a precision of 99%.

## 5 Conclusion

Cervical cancer is one of the deadliest and most common forms of cancer that affects women all over the world, but mainly in East Africa and South Asia. In addition, it is one of the tumours that affects women at the highest rate. The number of people diagnosed with cervical cancer has been steadily declining over the course of the last several decades in industrialised countries. In contrast, the rate of incidence decline has been much more gradual in emerging nations; indeed, there is some evidence to suggest that the incidence rate may even be rising in certain cases. To safeguard the health of women, it is imperative that tests for cervical cancer become standard practise and become part of regular healthcare. Due to the sluggish pace at which it spreads and develops into more severe stages of the illness, cervical cancer is notoriously difficult to identify and treat until it has progressed to an advanced stage. The screening for cervical cancer known as a Pap smear, which is more often referred to as a Pap test, offers the ability to identify the condition in its earliest stages. There is a multi-step process that must be completed before one may detect anything and then decide what that detection means. A method called a GLCM was used for the aim of picking characteristics for this piece of writing. After completing this stage, the classification process is carried out using techniques such as CNN, SVM, and auto encoder. The results of this experiment demonstrated that the GLCM-CNN classifier was the one that achieved the best level of accuracy.
